# Should we use cells, biomaterials, or tissue engineering for cartilage regeneration?

**DOI:** 10.1186/s13287-016-0314-3

**Published:** 2016-04-18

**Authors:** Jonathan C. Bernhard, Gordana Vunjak-Novakovic

**Affiliations:** Department of Biomedical Engineering, Columbia University, 622 West 168th Street, VC12-234, New York, NY 10032 USA; Department of Medicine, Columbia University, 622 West 168th Street, VC12-234, New York, NY 10032 USA

**Keywords:** Cartilage, Tissue engineering, Chondrocytes, Mesenchymal stem cells, Biomaterial, Bioreactor, Hydrogel, Implantation

## Abstract

For a long time, cartilage has been a major focus of the whole field of tissue engineering, both because of the constantly growing need for more effective options for joint repair and the expectation that this apparently simple tissue will be easy to engineer. After several decades, cartilage regeneration has proven to be anything but easy. With gratifying progress in our understanding of the factors governing cartilage development and function, and cell therapy being successfully used for several decades, there is still a lot to do. We lack reliable methods to generate durable articular cartilage that would resemble the original tissue lost to injury or disease. The question posed here is whether the answer would come from the methods using cells, biomaterials, or tissue engineering. We present a concise review of some of the most meritorious efforts in each area, and propose that the solution will most likely emerge from the ongoing attempts to recapitulate certain aspects of native cartilage development. While an ideal recipe for cartilage regeneration is yet to be formulated, we believe that it will contain cell, biomaterial, and tissue engineering approaches, blended into an effective method for seamless repair of articular cartilage.

## Background

Articular cartilage is a unique tissue that contains only a sparse population of a single cell type—chondrocyte—and lacks vascularization. The cells reside within a prestressed collagen–proteoglycan matrix that gives the tissue its compressive strength and enables frictionless motion during habitual loading. These features also severely hinder the ability of cartilage to regenerate after injury. Whether the injury is due to trauma or disease, and even if the lesion is relatively small, it can progress rapidly and lead to the destruction of cartilage structure and thereby its mechanical function. Because of the absence of self-repair, various interventions have been explored to facilitate regeneration of cells and cartilaginous matrix.

Traditionally, cartilage repair has been pursued by application of two main treatment methods, both of which have drawbacks. If cartilage is severely damaged so that the majority of the articulating surface is disabled, whole joint surgery can be performed where the living biological tissue is replaced with a prosthetic device. While these surgeries are quite successful and provide many years of joint function, the synthetic material cannot fully substitute for the complex biological nature of the original tissue. If cartilage injury is small and localized, an autograft or allograft can be trimmed to size and fit into the defect. However, with the cartilage properties being location dependent and because of the scarcity of cells that can facilitate graft integration with the host tissues, these grafting solutions often provide a limited-term benefit before ultimately failing [1]. A definite need therefore exists for more effective methods to stimulate cartilage regeneration and integration, and provide a durable, long-lasting replacement for the original cartilage.

**Fig. 1:**
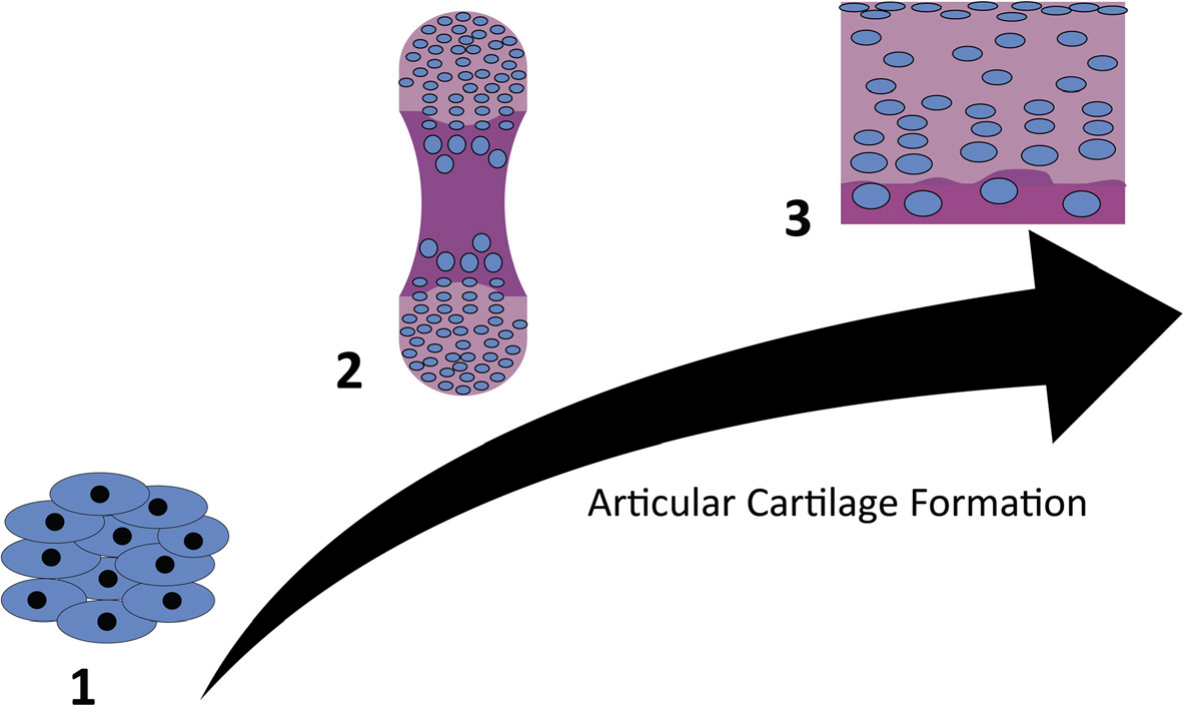
Chondrogenic development. (*1*) Mesenchymal condensation. (*2*) Chondrogenic differentiation of mesenchymal cells and deposition of a cartilage anlage that forms both cartilage and bone. (*3*) Remodeling of the anlage to form bone and mature cartilage with their inherent structural zones. Cartilage regeneration therapies have followed this approach, having investigated cellular therapies to trigger cartilage formation, biomaterial scaffolds that infiltrating cells can remodel, and tissue-engineered cartilage constructs that mimic the structure and function of native articular cartilage

In response to this need, novel bioengineering approaches to induce and enhance cartilage regeneration are being developed. When placed into the context of natural cartilage development, these approaches are based on achieving different landmarks in the process of cartilage formation, with the aim to recapitulate the developmental blueprints [2]. It is well established that cartilage formation begins with mesenchymal condensation leading to chondrogenic differentiation of mesenchymal cells. Then, a dense matrix is produced, serving as the cartilage anlage, a template for the subsequent generation of both the articular cartilage and the subchondral bone. The articular chondrocytes maintain their stable phenotype within mature articular cartilage (Fig. 1).

Scientists and engineers have attempted to develop biological grafts for treating cartilage defects by (*i*) cell therapies that recapitulate precartilaginous mesenchymal condensation and stimulate/orchestrate regeneration of cartilage, (*ii*) biomaterial matrices designed to restore important functions of articular cartilage and serve as a template for regenerative turnover, or (*iii*) tissue-engineered grafts for implantation that resemble mature cartilage and have capacity to integrate with the surrounding tissues [3, 4]. In this concise review, we present a brief survey of the merits of current research for each of these three approaches to cartilage regeneration, along with the status of their translation into the clinic, towards an initial response to the question posed about the prospects of using cells, biomaterials, or tissue engineering for cartilage regeneration.

### Cell therapies

Cells are the driving force of cartilage formation and the continual maintenance of the tissue. Cell therapies utilize the implantation of externally cultivated cells to replicate and stimulate native regeneration. Mature chondrocytes were the first cells that found clinical application for cartilage regeneration. Similar to autografts and allografts, the use of mature chondrocytes is based on the premise that native, mature cells are best suited to guide regeneration.

Mature chondrocytes in cell therapy applications have been primarily utilized in a technique termed autologous chondrocyte implantation (ACI). ACI starts by harvesting and enzymatically isolating chondrocytes from a minor load-bearing area of the patient’s damaged cartilage [5]. As cartilage has low cell density, the isolated chondrocytes have to be expanded in vitro to obtain enough cells for effective treatment. In ACI therapy, a membrane is placed over the defect that is filled with a suspension of chondrocytes and sutured to the surrounding cartilage to ensure chondrocyte localization within the defect [6]. Initially, a piece of periosteum cut out from the patient’s bone was used as a membrane, probably further contributing to cartilage regeneration as an additional source of cells. Later, synthetic membranes made of a collagen I/III blend were also used.

While the clinical trials reported good-to-excellent outcomes for almost all of the patients at 66 months, further randomized clinical trials demonstrated that ACI performed no better than microfracture surgeries [7]. Some limitations were also observed. When expanded on plastic in vitro, harvested articular chondrocytes tend to dedifferentiate and start producing substantial amounts of collagen type I. Such expansion and dedifferentiation can hinder hyaline cartilage formation and result in hypertrophic chondrocyte differentiation when implanted back into the defect [8]. Consequently, it has been noted that the cartilage formed following a traditional ACI procedure tends to histologically resemble fibrocartilage rather than articular cartilage [1]. Fibrocartilage is a dense, fibrous version of cartilage, lacking the compressive strength and frictionless properties of hyaline cartilage. The presence of fibrocartilage causes similar problems to the transplantation of articular cartilage autografts, where a lack of integration and mismatch in functional properties limit complete regeneration of the defect. Over the years, ACI has undergone numerous improvements [9], which have moved the ACI procedure from being classified as a cell therapy towards being a tissue-engineering therapy.

The use of mesenchymal stem cells (MSC) can alleviate two fundamental limitations of autologous chondrocytes: donor-site morbidity and limited matrix production following cell expansion. MSCs can be harvested from a number of sources that do not affect cartilage activity (a complete list is provided in [10]), maintain multipotency after numerous expansions, and can be differentiated into matrix-producing chondrocytes [11]. In addition, MSCs have immunomodulatory properties and have been shown to suppress proinflammatory cytokines [12]. However, it is currently being debated whether chondrogenically differentiated MSCs are programmed to progress towards terminal differentiation and bone formation [13, 14]. Primarily due to their favorable properties, and despite the current debate, MSCs are increasingly studied and utilized to treat cartilage defects and osteoarthritis [10].

For cartilage defects, the application of MSCs is similar to the ACI method, and has produced similar results. In a comparison of the procedures based on ACI and MSCs, there was no significant difference in the clinical outcome [15]. While the results of the ACI-type therapy using MSCs are promising, problems similar to those observed in the traditional ACI treatment still persist. Importantly, MSCs are a heterogeneous cell population that can generate fibrocartilage and hypertrophic chondrocytes along with the desired articular-cartilage-producing chondrocytes [16]. Studies have shown nonarticular cartilage being formed within the defect after implantation, and this situation was associated with poor clinical outcomes [17].

In attempts to more consistently derive articular chondrocytes that produce matrix and regenerate cartilage, investigators have turned to pluripotent stem cell sources: embryonic stem cells (ESCs) and induced pluripotent stem cells (iPSCs). While the use of ESCs is highly debated because of the ethics surrounding their derivation, iPSCs provide similar pluripotency without the ethical conundrum. In addition, iPSCs can be autologous as they can be derived from small samples of various tissues, including skin and blood. The derivation of chondrocytes from ESCs or iPSCs can be achieved by first deriving MSCs [18, 19], or by differentiating the cells directly into chondrocytes [20, 21]. Overall, the use of pluripotent stem cells for cartilage regeneration is highly promising. In comparison with MSC-derived chondrocytes, chondrocytes differentiated from pluripotent stem cells had higher gene expression for chondrocyte and cartilage-producing genes (*COL2A1*, *AGC*, *SOX9*), and reduced expression of hypertrophic and bone-producing genes (*COL10A1*, *COL1A1*, *RUNX2*) [22]. Numerous studies have shown the ability of these cells to produce articular-like cartilage that integrated with the native cartilage and subchondral bone when implanted into an animal defect [9, 22–24].

The ability of pluripotent stem cells to differentiate into all three germ layers and recapitulate the native, cartilage-producing cell phenotype is highly attractive for cartilage regeneration, but key challenges exist in efficiently producing and safely controlling these cells. Although recent advances have improved the efficiency of iPSC generation [25], the yields remain low for mass production. Also, the implantation of pluripotent stem cells has occasionally resulted in teratoma formation [26, 27]. Studies have shown that genetic disorders, such as Marfan’s syndrome, still manifest in derived chondrocytes, even after the induction of pluripotency and subsequent in-vitro differentiation [28]. Despite promising results in stimulating cartilage regeneration, the pluripotent stem cell generation, the inability to recover genetic deficiency, and the risk of teratoma formation pose significant questions that must be addressed before clinical translation.

While major progress has been made using each of the cell types discussed, it is still being investigated how to overcome the current limitations and produce viable, functional, and durable cartilage capable of integrating with the surrounding tissues. Clinical trials of cartilage therapies using pluripotent stem cells (either embryonic or derived from adult human tissues) have been slowed down by concerns that multipotent stem cells have potential to form teratomas [9]. In clinical practice, ACI is a well-established cell therapy for cartilage repair, with variations of the procedure used in the clinic since 1987, and numerous revisions and improvements [29]. The use of MSCs for cartilage therapy is currently undergoing the rigors of regulatory approval, with the first clinical study performed in 2004 [10], providing cartilage repair without the need to harvest chondrocytes from the patient.

### Biomaterials

Because of the seemingly quiescent nature of cartilage, its avascularity, and its low cell density (only about 5 % of the tissue volume), a significant branch of research has been directed towards developing biomaterials that can mimic cartilage matrix and restore function at the defect site. In particular, the biomaterials of choice must meet three significant criteria: (*i*) mechanical properties consistent with those of existing cartilage (both in terms of compressive strength and lack of friction), (*ii*) integration with adjacent cartilage, and (*iii*) durability throughout the patient’s lifetime [30]. Currently used biomaterials are in general biodegradable, with the goal that the biomaterial will eventually be eliminated from the body after providing the necessary functional properties to support cartilage regeneration. For many materials, the rate and mechanisms of biodegradation are tunable, which is important for matching the kinetics of biomaterial resorption and tissue formation.

Up to 80 % of articular cartilage wet weight consists of water, its most abundant component [31]. To replicate this environment, hydrogels—three-dimensional (3D) polymer networks rich in water—have become a popular option for cartilage regeneration in situ and cartilage engineering in vitro. Hydrogels are highly modular, with respect to the type of polymer, the crosslinking method, the degradation products and rate, and the incorporation of various molecules, all allowing specific tailoring for the desired application [32, 33]. Hydrogels are classified based on the polymer composition into natural or synthetic materials, although combination systems are also being used to maximize the benefits of each component. We will briefly touch on the most popular polymers from each group.

Natural polymers can be derived from both animal and plant sources. Three of the most popular polymers that are used as hydrogels are alginate, agarose, and silk, with the first two derived from seaweed and the last derived from either silkworms or spiders [32]. The unique composition of these polymers makes them unrecognizable to human enzymes, allowing slow degradation and more time for the body to initiate and support regeneration [32]. The mechanical properties of these hydrogels can be adjusted using the right formulations and postprocessing methods [33]. In addition, these hydrogels have been shown in vitro to provide suitable environments to maintain the phenotype of encapsulated chondrocytes. However, these particular hydrogels lack natural attachment sites for cells and inherent bioactivity to trigger synthesis of extracellular matrix (ECM) [23, 34]. Although readily available, semicustomizable, and easy to work worth, the ability of these scaffolds to stimulate regeneration and integrate in an in-vivo repair situation is yet to be determined conclusively.

Collagen and hyaluronan are the two popular natural polymers that are recognizable by mammalian cells and, at the same time, are important components of native articular cartilage. Collagen, as the most common protein in the body, has been extensively studied for cartilage regeneration [35]. Collagen has a natural tendency to support cell attachment and stimulate synthesis and assembly of the ECM. However, when implanted, collagen hydrogels are mechanically weaker than the surrounding tissue and degrade too fast relative to tissue regeneration [36, 37]. Crosslinking of the collagen matrices can improve their mechanical integrity and slow down degradation, but it also can have significant impact on the encapsulated cells [38].

In native cartilage, chondrocytes surround themselves with a hyaluronan-based pericellular matrix, which has led many investigators to designing hydrogels consisting of hyaluronan [39]. As expected, chondrocytes readily attach to hyaluronan-based matrices, and studies have shown that these matrices trigger chondrocyte differentiation and stimulate matrix production [40, 41]. Hyaluronan also has some limitations, such as insufficient mechanical integrity and a short lifetime in inflamed defects due to degradation by matrix metalloproteinases [42].

In contrast to natural polymers, synthetic polymers provide a high level of control of compositional, structural, and mechanical properties. Polyglycolic acid (PGA) and polylactic acid (PLA) have gained particular interest because they degrade by simple hydrolysis at rates that can be adjusted by selecting monomers, and have already been approved for clinical use as sutures [43]. A major drawback of synthetic polymers is that they do not provide specific biological functions [44]. To facilitate cell attachment and stimulate matrix production, the synthetic polymers need to be functionalized with biological motifs or bioactive molecules [45].

Polymer modifications are widely utilized to control cell activities within the body. Of particular interest are efforts to recruit native cells and promote their differentiation and regenerative capabilities [43, 46]. The incorporation of growth factors and biomolecules, such as dexamethasone and transforming growth factor beta, has shown promise for facilitating cartilage regeneration [47, 48]. Such functionalized scaffolds release the incorporated modulatory components as they are degraded, and can thereby enhance cartilage regeneration within a defect. However, this interesting approach has not been fully realized, and the degree of regeneration achieved thus far has not been convincing enough to justify clinical translation.

An important trend in surgery, both general and orthopedic, is to minimize the severity of intervention [49]. The use of an arthroscopic procedure instead of open joint surgery can reduce the infection risk and shorten the time of recovery. For cartilage repair, the use of injectable hydrogels is of special interest because they are compatible with arthroscopic methods. By introducing the regenerative hydrogel into the defect by injection through the joint capsule, procedural severity and duration as well as the duration of recovery are reduced significantly [49]. Many of these polymers have been transitioned into injectable formats. The two most common forms of initiating the transition from injectable liquid to hydrogel are thermal activation, which results in the crosslinking of hydrogel at body temperature, and light activation, which initiates crosslinking of hydrogel in the presence of a specific light source [44, 50, 51].

Beyond any doubt, biomaterials will continue to be a major focus of research for cartilage regeneration. For clinical purposes, biomaterials are extremely attractive because they can be utilized off-the-shelf, have an established precedence of clinical use, and have much simpler and shorter regulatory procedures than cell-based products.

### Tissue engineering

The incorporation of cells into biomaterial scaffolds makes cartilage repair more complex, but can significantly help orchestrate regeneration and overcome some of the limitations of using cells or biomaterials alone. After several decades of research in cartilage regeneration, either through cell therapy or biomaterial implantation, we still lack robust methods for reestablishing durable articular surfaces with mature functional tissue properties. In an effort to address this major challenge, scientists and engineers turned to tissue engineering methods designed to replicate the developmental steps of cartilage formation.

In general, tissue engineering combines cells and biomaterial scaffolds into a tissue construct, and then uses engineered control of the construct environment, in vitro and in vivo, to replicate the native cartilage environment and produce viable grafts for cartilage regeneration [52]. Here we focus on the inclusion of additional, external engineered controls to create these cartilage constructs. Mechanical stimulation, oxygen tension, and 3D printing are among the methods that have been utilized to replicate the in-vivo environment supporting chondrocyte differentiation and matrix production.

Articular cartilage is subjected to cyclic forces and the application of physiological levels and regimens of stress is considered to be essential for chondrocyte viability and function [53]. To replicate this environment, compressive forces were applied to tissue-engineered constructs during in-vitro cultivation. Specially designed bioreactors were constructed that allowed control of the rate and amplitude of the applied stress [53]. Dynamic mechanical compression has been shown to trigger chondrocyte differentiation [45], stimulate matrix production [54], and increase cell viability and proliferation [23]. These beneficial effects are presumably due to a combination of direct mechanical factors (and their transduction into gene expression) and enhancement of mass transport by compression-induced interstitial flow. Constructs that were conditioned within bioreactors with mechanical loading approached more closely the compressive properties of native cartilage, as the application of force dictated the location and alignment of matrix deposition by resident chondrocytes [55, 56]. The application of compressive forces to the tissue-engineered construct could be utilized to mature and strengthen the graft, signaling the chondrocytes to replicate a cartilage-mimicking structure.

Studies of the dynamic environment of cartilage revealed that the individual chondrocytes did not actually experience direct compressive stress, but rather a hydrostatic pressure caused by the swelling and fluid movement associated with the articular cartilage loading [53]. Hydrostatic bioreactors were therefore constructed to replicate the in-vivo environment in a loaded joint [57], producing similar results to those achieved in response to compressive loading. Both the chondrocytes and MSCs expressed higher levels of cartilage genes and produced larger amounts of glycosaminoglycans and type II collagen [58, 59].

The environment of healthy cartilage is hypoxic, with oxygen tensions ranging between 1 and 5 %, as compared with 21 % in ambient air [53]. However, traditional tissue culture is in most cases carried out in normoxic conditions. Lowering oxygen tension has enhanced MSC and chondrocyte differentiation and arrested hypertrophic maturation [60–62]. There is still a debate about the degree of ECM synthesis stimulated by hypoxia, as conflicting reports have been published on both sides of the argument [63]. However, as has been shown with both the mechanical stimulation and oxygen tension, replicating the native environmental conditions of cartilage has beneficial effects on engineered cartilage.

3D printing has also gained a considerable amount of attention due to its ability to provide precise control of the initial structure of tissue-engineered constructs. While other processing methods have been used previously to mimic the structure of native cartilage [64], the novelty of 3D printing and the high degree of precision (almost to the cellular level) are the focus in this review. An excellent recent review by Garg and Goyal [64] covers the broad scope of fabrication processes. With regards to cartilage, ongoing work indicates that 3D printing has the potential to replicate the specific structure of cartilage, depositing an appropriate pericellular environment for the cells located in each cartilage zone [65–67]. The most successful studies to date have recapitulated an osteochondral defect, using different substrates and patterns for bone and cartilage components [68, 69]. Bioprinting directly into a created, ex-vivo cartilage defect resulted in some level of integration into the native cartilage and mechanical competence [70].

Despite the increased ability of tissue engineering to mimic the native environment and enhance cartilage matrix production, current tissue constructs are still not stratified and mechanically functional, and therefore are not suitable for clinical use [71]. Carticel, a longstanding tissue-engineered product, has resulted in the development of articular cartilage that integrated with the adjacent native cartilage. However, this method requires harvesting the chondrocytes from the patient’s knee, resulting in tissue morbidity and the need for two surgeries. In contrast, MSCs from bone marrow or fat aspirates are much easier to harvest, can be expanded in culture, and can be differentiated into chondrocytes. However, stratified structure and mechanical function have not been achieved with these cells. Interestingly, the work by Mauck et al. [72] shows that these limitations are not a result of delayed cell differentiation, but are due to a missing link in the development of cartilage tissue-engineered constructs.

**Fig. 2:**
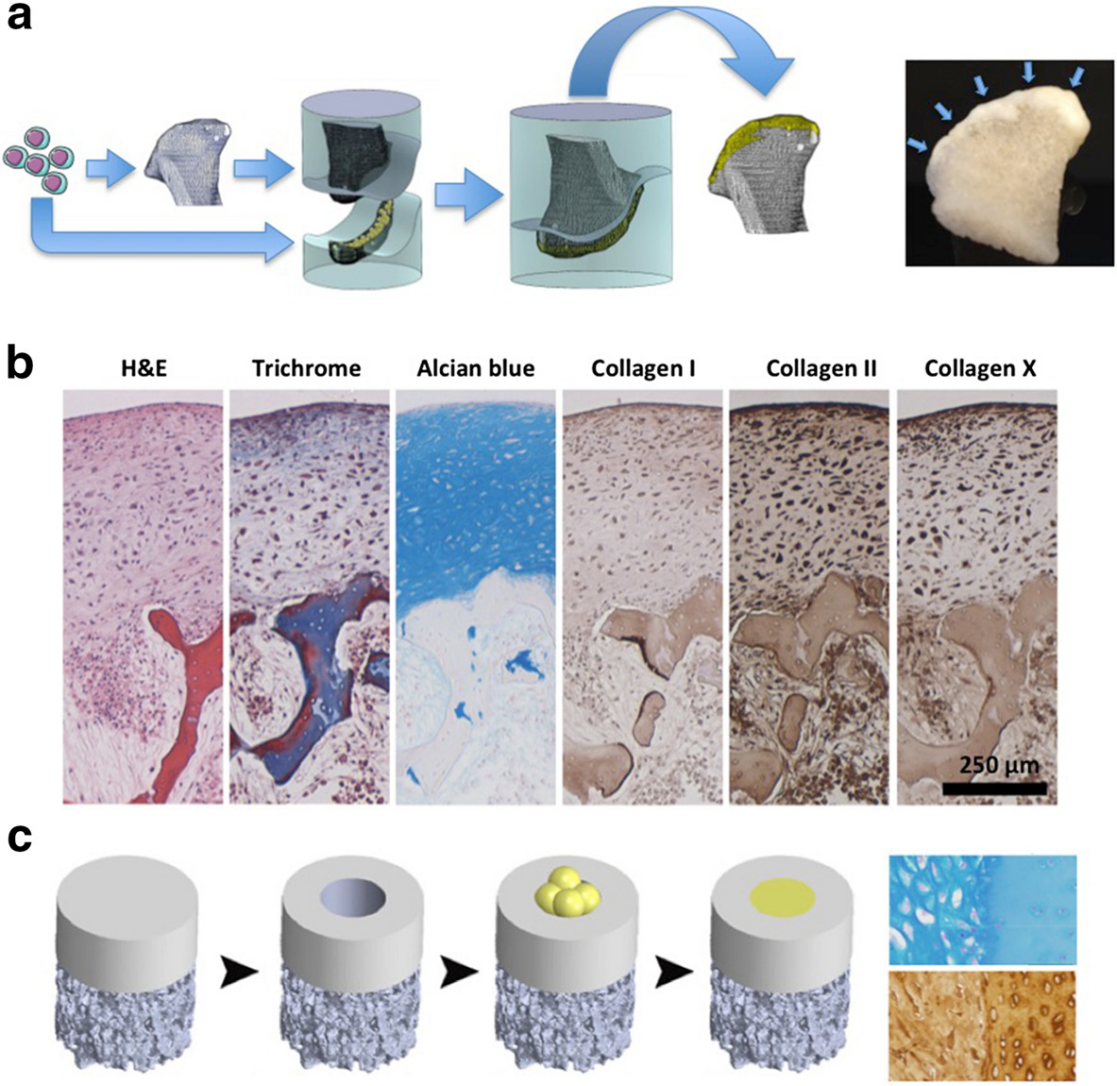
Engineering of stratified, mechanically functional human cartilage. **a** Human mesenchymal stem cells are induced to fuse into cell bodies which are then placed on the cartilage side of a mold in the exact shape of a condyle, an anatomically shaped bone scaffold is placed on the other side, and the two pieces are press-fit. After 5 weeks of in-vitro cultivation, an anatomical layer of articular cartilage forms at the interface with the underlying bone. **b** The resulting cartilage is physiologically thick and stratified, expressing all key markers, and integrated with the underlying bone. **c** The fusing mesenchymal stem cell bodies were also tested for their ability to repair small cartilage defects. Structural integration is shown by alcian blue and antibody stains for glycosaminoglycan and collagen type II. The newly formed tissue is shown on the *left*, the adjacent native cartilage on the *right*. Selected images are reproduced with permission from [73]. *H & E* hematoxylin and eosin

A novel approach that appears to find this missing link recapitulates important steps in the native development of cartilage (Fig. 2). In these studies, chondrocytes and stem cells were condensed into scaffold-less constructs that formed cartilaginous tissue in a manner mimicking mesenchymal condensation that precedes native chondrogenesis [73–76]. This condensation-based approach demonstrated promising results, with studies revealing structural similarity of the tissue-engineered construct to native cartilage [73–76]. In one approach, biomechanical properties of cartilage derived from condensed human mesenchymal cells were comparable with native cartilage, with Young’s modulus of approximately 850 kPa and equilibrium friction coefficient <0.3. This method also formed mechanically strong cartilage and an interface in a cartilage defect model [73]. The mimicking of native development of cartilage with application of external controls provides an enticing way forward in the elusive pursuit of a translatable cartilage-defect solution.

## Conclusions

We still lack reliable methods to generate durable articular cartilage that would resemble the original tissue lost to injury or disease. For clinical translation, a product that is available off-the-shelf, can be applied without surgery, integrates seamlessly into the native cartilage, and incorporates native cells to allow remodeling would be most highly desired. Basic and translational studies conducted over the last several decades markedly advanced our understanding of cartilage development, normal function, and pathological function.

Cartilage has proven to be both simple (with its sparse population of a single cell type, absence of vascular supply) and very complex (as its prestressed matrix and its structural and mechanical properties are rather difficult to engineer). The question posed here is whether cartilage regeneration will be achieved using cells alone, or biomaterials, or tissue engineering. We propose that the ultimate therapeutic modalities will actually combine the best elements of all three approaches. We also propose that the overriding principle for the development of effective clinical modalities will be in the recapitulation of some of the key steps in native cartilage development, such as the early steps of mesenchymal condensation and the development of cartilage anlage. The simplest and most robust method for achieving durable cartilage repair will certainly have the highest chance of clinical acceptance. The field will need to determine how simple is complex enough, how much needs to be done in vitro prior to implantation, whether inflammatory responses can be harnessed to enhance regeneration, and how to achieve integrative repair in a diseased joint.

## Abbreviations

3D: three-dimensional
ACI: autologous chondrocyte implantation
ECM: extracellular matrix
ESC: embryonic stem cell
iPSC: induced pluripotent stem cell
MSC: mesenchymal stem cell
PGA: polyglycolic acid
PLA: polylactic acid
